# Facial Nerve Palsy With Olfactory and Gustatory Dysfunction Following COVID-19 Infection: An Illustrative Case-Based Narrative Review

**DOI:** 10.7759/cureus.116106

**Published:** 2026-09-11

**Authors:** Sushant Jain

**Affiliations:** 1 Research, Global Medical Education Institute and Publishing, New York, USA

**Keywords:** anosmia, covid-19, dysgeusia, facial nerve palsy, neuroinvasion, olfactory nerve dysfunction, pandemic, sars-cov-2

## Abstract

COVID-19 infection has been recognized to induce a wide range of neurological manifestations, ranging in severity from transient, benign manifestations to severe neurological deficits. Cranial nerve involvement, particularly facial, olfactory, and gustatory nerve dysfunction, has emerged as a notable clinical feature in affected individuals. This review article aims to present and analyze three confirmed COVID-19 diagnoses that exhibited facial nerve palsy (FNP), olfactory disturbances (OD), and/or gustatory disturbances (GD), while also exploring the potential underlying pathophysiological mechanisms of these symptoms. This article presents three cases of laboratory-confirmed COVID-19 in which patients developed FNP along with OD and/or GD, and discusses their potential pathophysiological mechanisms. All three patients had hallmark signs and symptoms of FNP, with dysgeusia present in three and anosmia present in two. COVID-19 was confirmed with reverse transcription polymerase chain reaction (RT-PCR) testing. Neuroimaging and cerebrospinal fluid analysis ruled out other differential diagnostic possibilities. Treatment strategies varied from case to case and included steroids, antivirals, and traditional medicine. Outcomes ranged from partial to complete recovery of FNP within four weeks. The three presented cases suggest a temporal association between COVID-19 and FNP, though case reports alone cannot establish causation. While direct neural invasion, immune response, or ischemic injury are potential mechanisms, a definitive cause has not yet been established. OD and GD in the presented cases were short-lived, which may suggest, but cannot confirm, a mechanism different from direct cranial nerve involvement. This remains speculative given the small sample size. Further research is warranted to elucidate the pathophysiology of cranial nerve involvement, thereby guiding diagnosis and treatment strategies.

## Introduction and background

The COVID-19 pandemic is the most consequential acute illness of the 21st century, with more than 775 million confirmed infections and over seven million deaths worldwide [1]. It has profoundly impacted the medical landscape and reshaped our lives, from family and social interactions to education to world economics. The causative organism was sequenced, identified as a novel virus, and named severe acute respiratory syndrome coronavirus 2 (SARS-CoV-2) [2].

Although an acute respiratory illness typically presents with fever, cough, fatigue, and dyspnea [3,4], SARS-CoV-2 can also infect many other tissue types by using or attaching to angiotensin-converting enzyme 2 (ACE2) as the receptor for cellular entry [5,6]. Because ACE2 is widely expressed throughout the body, including in cells of the central nervous system (CNS) and peripheral nervous system (PNS), the virus can affect a wide range of tissues [7,8]. Neurological symptoms may occur as direct effects of SARS-CoV-2 neurotropism on the CNS and PNS, or as systemic consequences of a para-infectious or post-infectious immune-mediated mechanism [9]. The CNS sequelae included headache, dizziness, stroke, intracerebral hemorrhage, encephalitis, acute myelitis, and encephalopathy, which can lead to altered mental status and delirium, as well as seizures and neuropsychiatric symptoms. The PNS involvement includes Guillain-Barré syndrome (GBS) and its variants, olfactory dysfunction (OD, including hyposmia and anosmia), gustatory dysfunction (GD, including dysgeusia and ageusia), cranial nerve palsy, autonomic nervous system complications, neuralgia, and ocular muscle abnormalities [10,11]. Consequently, it can lead to long COVID, or post-COVID condition (PCC), which refers to a constellation of new or persisting symptoms that appear or continue after the acute phase of a SARS-CoV-2 infection and typically last four or more weeks. Neurological symptoms of PCC include brain fog, headaches, tremors, OD, GD, insomnia, and tinnitus, to name a few [12].

The chaotic nature of the COVID-19 pandemic meant that patient recruitment for clinical studies and evaluations was often arduous and limiting, with significant differences between variables such as patient location and COVID-19 severity. This is reflected in the wide variance in the reported prevalence of neurological presentations in COVID-19 [13]. The pooled prevalence with 95% confidence interval of various neurological manifestations reported in the COVID-19 patients was found to be headache 14.6% (12.2-17.2), OD 26.4% (21.8-31.3), GD 27.2% (22.3-32.3), GBS 6.9% (2.3-13.7), etc., with high heterogeneity among the studies [14] (Figure 1). Given this heterogeneity, these pooled estimates should be interpreted as approximate ranges reflecting variability in patient populations, disease severity, and case-ascertainment methods across the included studies, rather than as precise, generalizable figures.

**Figure 1:**
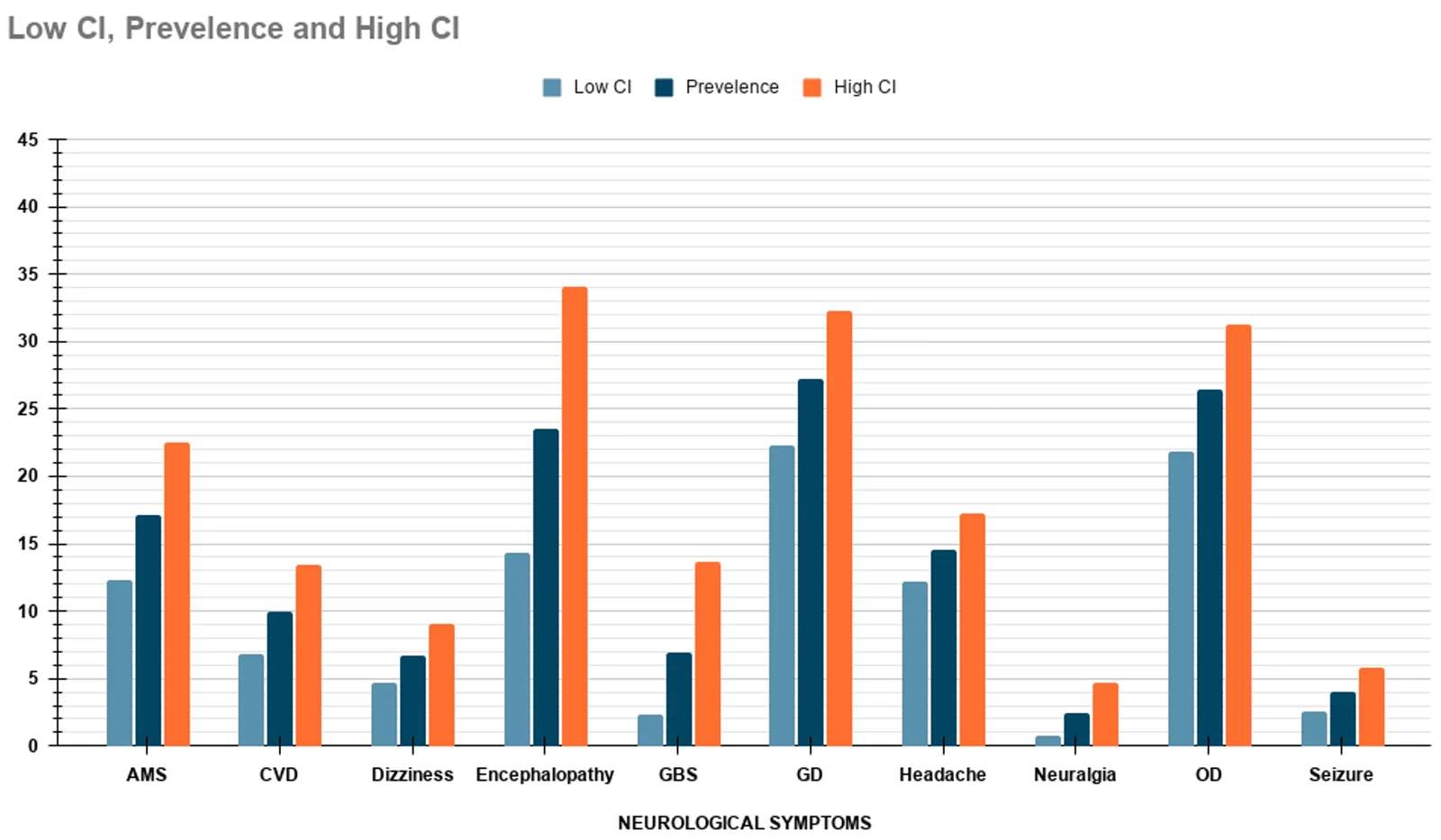
Prevalence of neurological symptoms in COVID-19 cases. Low CI: lower limit of 95% confidence interval; High CI: upper limit of 95% confidence interval; AMS: altered mental status; CVD: cerebrovascular disease; GBS: Guillain-Barré syndrome; GD: gustatory dysfunction; OD: olfactory dysfunction. Adapted from Ref. [14].

The discussion on neuroinvasion surrounds various proposed routes of viral entry into the CNS, with the two most published pathophysiologic mechanisms [15]. Direct evidence for these pathways in living human tissues is limited, and much of the evidence is derived from in vitro studies, animal models, and post-mortem tissue analysis [15]. 

The hematogenous route, in which the virus needs to spill over into the circulation (viremia) and subsequently cross the blood-brain barrier (BBB), blood-cerebrospinal fluid barrier [16], and/or enter the CNS via areas lacking BBB, such as the circumventricular organs (Figure 2).

**Figure 2:**
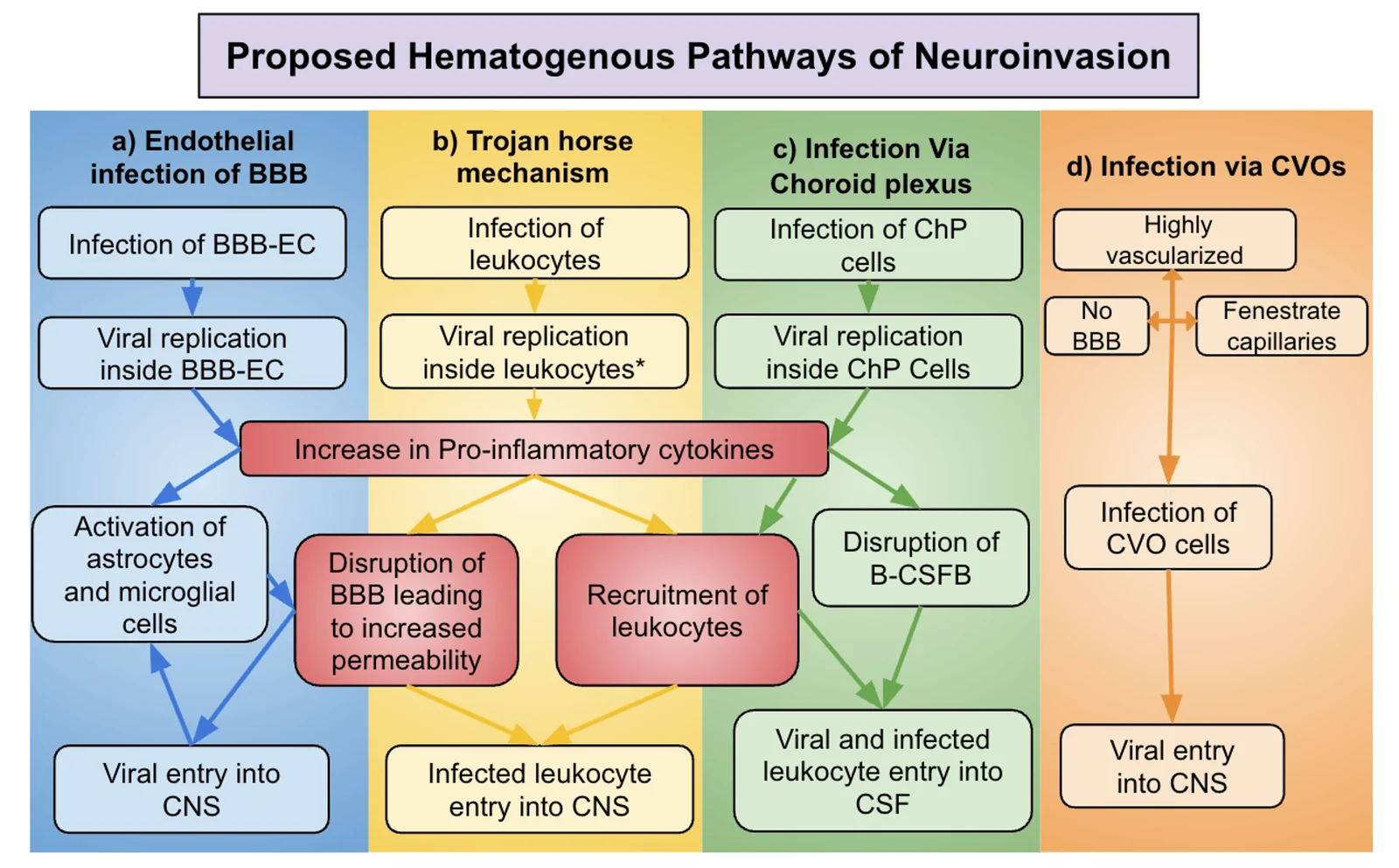
Proposed hematogenous pathways of neuroinvasion. BBB: blood-brain barrier; BBB-EC: blood-brain barrier endothelial cells; ChP: choroid plexus; B-CSFB: blood-cerebrospinal fluid barrier; CSF: cerebrospinal fluid; CNS: central nervous system; CVOs: circumventricular organs. Adapted from Refs. [17-21]. Image created using Google Docs (Google LLC, Mountain View, CA, USA).

The neural route of infection of peripheral nerves is proposed to be followed by Jagst et al. [15]: migration of SARS-CoV-2 via retrograde axonal transport (Figure 3a) and trans-synaptic spread (Figure 3b) to infect the brainstem [15,20,22] (Figure 3).

**Figure 3:**
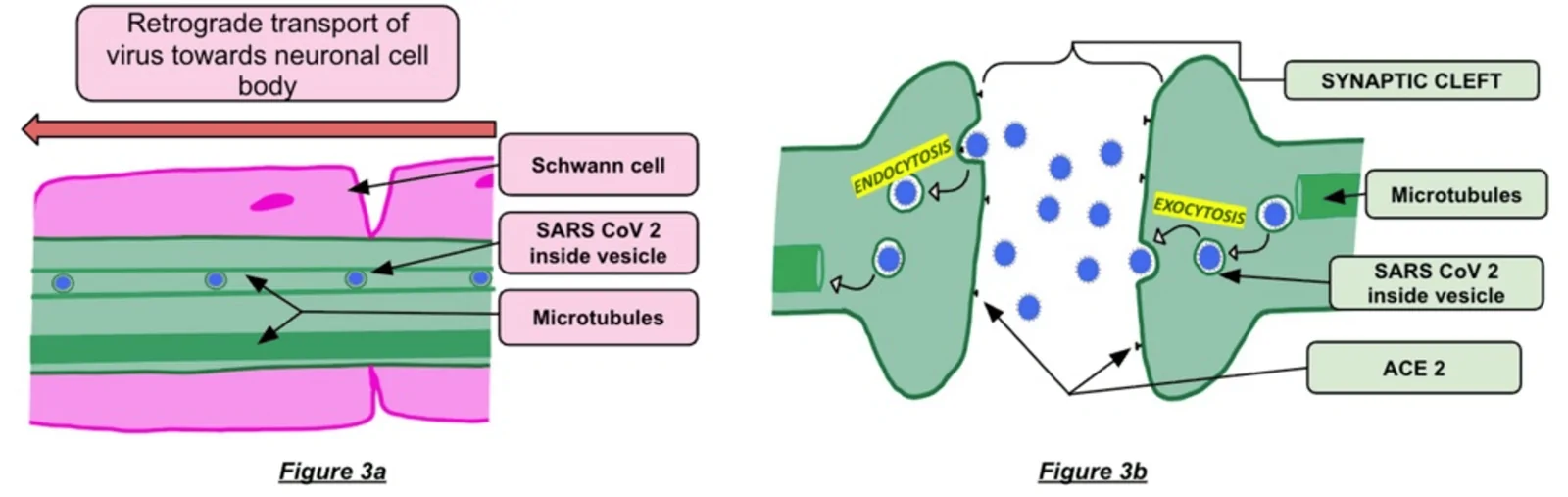
Potential neural pathway of SARS-CoV-2 neuroinvasion. (a) Retrograde axonal transport of SARS-CoV-2 via microtubules towards the neuronal cell body [15,20,23-25]. (b) Transsynaptic transfer of SARS-CoV-2 using vesicular exocytosis and endocytosis mechanisms [26]. SARS-CoV-2: severe acute respiratory syndrome coronavirus 2; ACE2: angiotensin-converting enzyme 2. Adapted from Refs. [15,20,23-26]. Image credit: Sushant Jain, created using Notability app (Ginger Labs, Inc., Silicon Valley, CA, USA) on an iPad.

Cranial nerve palsies are increasingly recognized in the literature as one of the neurological manifestations. SARS-CoV-2 can encounter cranial nerves at many points in the respiratory tract. For instance, the olfactory nerve (CN I) and ophthalmic branch of the trigeminal nerve (CN V1) are located in the nasal cavity, the facial nerve (CN VII) and glossopharyngeal nerve (CN IX) in the upper respiratory tract, and the vagus nerve (CN X) in the lower respiratory tract [15]. Multiple systematic reviews and studies have reported that CN VII is the most frequently involved cranial nerve, followed by either the vestibulocochlear nerve (CN VIII) or the olfactory nerve (CN I) [27-29]. However, one retrospective study with 98 patients found that the olfactory nerve was the most frequently involved cranial nerve, followed by CN VIII, with none of the 98 patients exhibiting CN VII involvement [30].

While COVID-19 can affect any cranial nerve and multiple nerves contemporaneously, the exact mechanism of cranial nerve palsy associated with SARS-CoV-2 infection is idiopathic. Although OD, GD, and FNP have each been well characterized individually among the neurological manifestations of COVID-19, their concurrent presentation as a single clinical triad remains comparatively underexamined, and it is unclear whether a shared or divergent pathophysiological mechanism underlies their co-occurrence. This review addresses this triad specifically, using three illustrative cases of FNP with concurrent olfactory and/or gustatory dysfunction in the setting of confirmed SARS-CoV-2 infection, confirmed by reverse transcription polymerase chain reaction (RT-PCR), to situate the presentation within the broader literature on cranial nerve involvement in COVID-19.

## Review

Methodology 

This is a narrative, illustrative review rather than a systematic review or meta-analysis. Its purpose is educational, to synthesize and illustrate, through well-documented cases, the clinical recognition, diagnostic workup, and proposed mechanisms of this symptom combination as described in the existing literature, rather than to report novel clinical or mechanistic findings. Cases were identified through a search of PubMed Central (PMC) and the Consensus academic search engine using combinations of the terms "COVID-19," "SARS-CoV-2," "facial nerve palsy," "facial paralysis," "neuropathy," "anosmia," "ageusia," and "dysgeusia," covering literature published from the emergence of SARS-CoV-2 in late 2019 through early 2025. Case reports were included if they described laboratory-confirmed SARS-CoV-2 infection with FNP accompanied by olfactory dysfunction, gustatory dysfunction, or both, and if sufficient diagnostic workup (imaging, CSF analysis, and/or serology) was reported to support the diagnosis. Reports lacking confirmatory testing or adequate clinical detail were excluded. The three cases presented here were the only published reports identified that met all inclusion criteria; no additional qualifying cases were excluded or set aside. Given the illustrative, case-based nature of this review, no formal risk-of-bias assessment or statistical synthesis (e.g., meta-analysis) was performed. Cases are synthesized narratively rather than through formal thematic coding or quantitative pooling.

Case 1 

A 64-year-old woman presented to the neurology department with a chief complaint of the abrupt onset of left hemifacial weakness. She denied any preceding constitutional symptoms except for a new-onset headache, but reported close contact with a patient who was hospitalized for COVID-19 pneumonitis. On admission, stable vitals were noted, and on physical exam, facial asymmetry, left eyebrow droop, impaired ability to wink, and numbness around the left nasolabial fold were noted. The rest of the physical and neurological exams were normal [31].

Her diagnostic tests, which included serologies for human immunodeficiency virus (HIV), herpes simplex virus (HSV), and varicella zoster virus (VZV), as well as brain computed tomography (CT) and magnetic resonance imaging (MRI), were normal; however, her lung CT revealed ground-glass opacities indicative of COVID-19, which RT-PCR confirmed for SARS-CoV-2. Her anti-SARS-CoV-2 IgM titers were also higher than the normal upper limit. Electromyography and nerve conduction velocity testing revealed an abnormality in the left facial nerve. A diagnosis of FNP in the setting of COVID-19 infection was made. The patient was prescribed lopinavir/ritonavir (Kaletra) 400 mg twice daily and dexamethasone 4 mg twice daily.

At the two-week follow-up, her facial weakness had improved significantly, and she had completely recovered her sense of smell and taste. One month later, her facial palsy improved but was not resolved, and her repeated nasopharyngeal COVID-19 RT-PCR was negative. The patient was then lost to follow-up.

Case 2 

A 35-year-old woman presented to the hospital in Japan with a two-day history of cough, malaise, sore throat, and nausea. She was recently aboard a cruise ship, traveling as an interpreter for the past five months. She had no significant medical history but was reported to be a habitual smoker [32]. 

At admission, she reported improvement in her sore throat but with fever, malaise, and a worsening cough. Her temperature was 39.8°C, her pulse was 110 beats per minute, and her other vital signs were stable. A physical exam revealed tenderness in the right neck, swollen tonsils, and right facial paralysis with loss of taste on the right side of her tongue. The patient also reported complete anosmia.

Rapid tests for influenza and streptococci returned negative. Radiographs of the chest revealed no abnormalities, but a CT scan of her lungs showed multiple bilateral ground-glass opacities. The nasopharyngeal collection returned positive for SARS-CoV-2. CBC revealed a low blood cell count of 3,320/mL (neutrophils 72%, lymphocytes 18.4%, basophils 0.3%, eosinophils 2.1%, and monocytes 7.2%) and an elevated C-reactive protein level of 2.81 mg/dL. The cerebrospinal fluid (CSF) analysis was normal and negative for the SARS-CoV-2 PCR assay. 

The diagnosis of COVID-19 pneumonia and associated FNP was made. The patient was initially treated only with Maoto (a traditional Japanese medicine known as Kampo). Still, fever and cough persisted after two days, but FNP, smell, and taste showed minor improvements. On the fifth day, her respiratory symptoms worsened, and she started inhaling ciclesonide. On the sixth day, favipiravir was administered, and fever and respiratory distress ceased to worsen. Significant improvements in facial paralysis, taste, and smell were also noted. The patient was asymptomatic by day 11. Repeat SARS-CoV-2 tests were negative on day sixteen, and she was discharged in stable condition.

Case 3 

A 27-year-old man was admitted to a tertiary health care center in Singapore with a four-day history of myalgia, cough, fever, and a new left-sided throbbing headache. The physical examination revealed bilateral conjunctival injection, but no neurological symptoms were noted. The chest X-ray did not reveal infiltrates or opacities, but the RT-PCR assay of the nasopharyngeal swab returned a positive result for SARS-CoV-2 [33]. 

On the third day of hospitalization, the patient developed dysgeusia and left retroauricular pain, followed by left facial weakness. A neurological exam revealed involvement of the left frontalis, orbicularis oculi, buccinator, and orbicularis oris muscles, which would lead to the inability to wrinkle the forehead, elevate the eyebrows, lagophthalmos, and droop at the corner of the mouth, respectively, consistent with FNP.

The rest of the neurologic examination was unremarkable, and his reflexes were normal. No associated vesicles in the outer ear or parotid swelling were noted. The HIV screen was negative. CSF analysis showed no pleocytosis, and glucose and protein levels were normal. CSF PCR for HSV, VZV, Epstein-Barr virus (EBV), cytomegalovirus (CMV), and RT-PCR for SARS-CoV-2 were negative. MRI revealed enhancement of the left facial nerve. Based on the new symptoms and high SARS-CoV-2 viral load, FNP was made in an active COVID-19 infection setting. 

The patient was on prednisone + valacyclovir for the FNP and lopinavir/ritonavir to reduce viral replication. There was no significant improvement in FNP at one week, but his headache and retroauricular pain showed improvement. By the second week, his viral load was undetectable.

Discussion

The three cases presented symptomology consistent with, but not diagnostic of, FNP associated with SARS-CoV-2 infection, demonstrating distinct neurological comorbidities of COVID-19. The hallmark signs and symptoms of FNP were observed in all three cases: eyebrow droop, ageusia/dysgeusia (loss of taste or altered sense of taste), and facial asymmetry. Notably, Cases 1 and 3 presented with headache and lagophthalmos (incomplete eyelid closure). Periauricular otalgia was unique to Case 3, and numbness of the nasolabial fold was unique to Case 1 (Figure 4).

**Figure 4:**
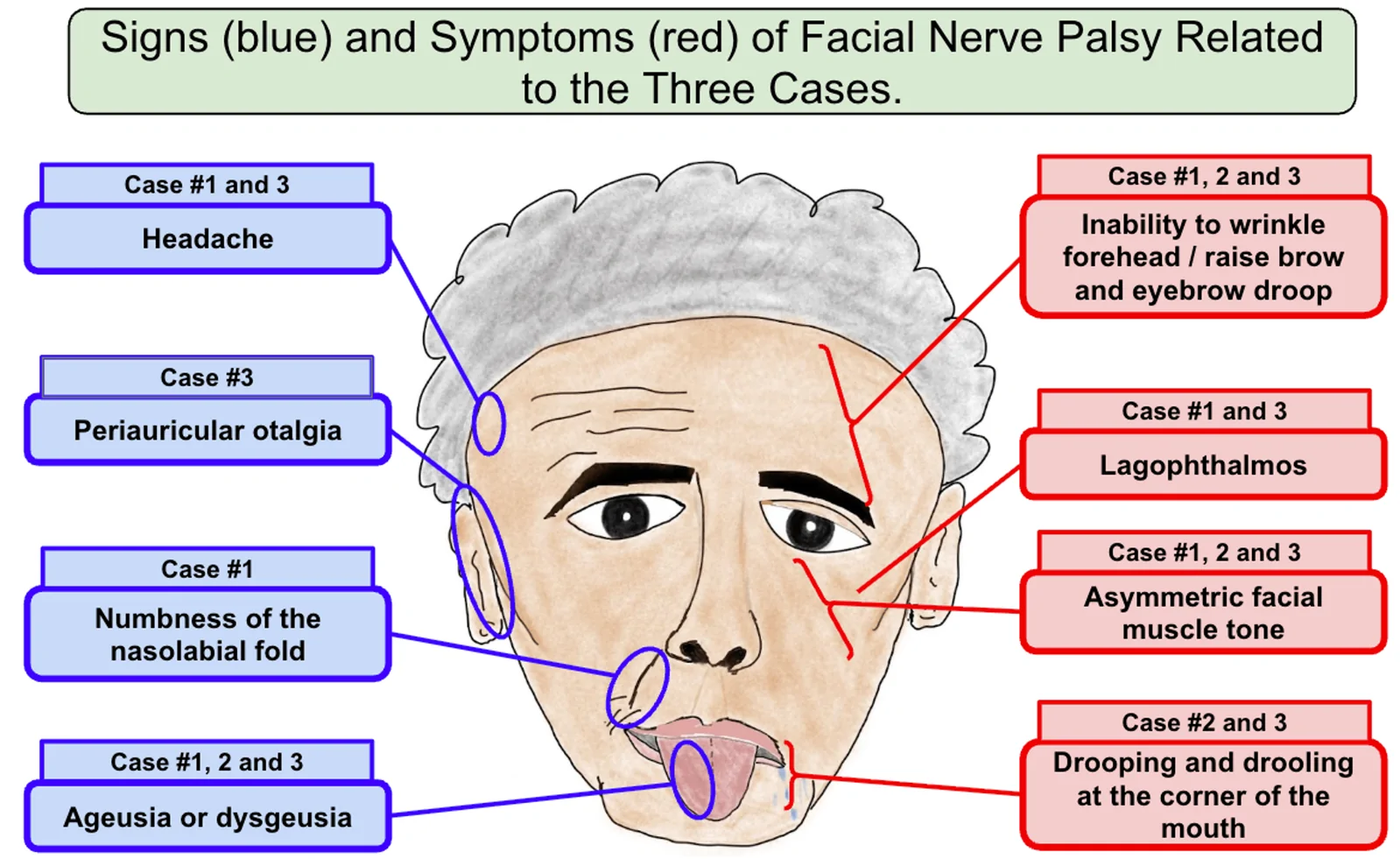
Signs (blue) and symptoms (red) of facial nerve palsy related to the three cases. Adapted from Refs. [34-36]. Image credit: Sushant Jain, created using Notability app (Ginger Labs, Inc., Silicon Valley, CA, USA) on an iPad.

Epidemiologic and diagnostic comparison

Epidemiologically, all three cases reported a history of exposure to high-risk environmental factors for SARS-CoV-2. Case 1 reported close contact with a hospitalized patient with COVID-19 pneumonitis. Case 2 reported employment as a translator on an international cruise ship, and Case 3 reported an international travel history.

Diagnostically, SARS-CoV-2 infection was confirmed by a positive RT-PCR in all three cases. Further laboratory evaluation in Case 1 revealed an anti-SARS-CoV-2 IgM level above the upper normal limit, but the rest of the serologies were normal. High viral load during the early phase, days 4-7 of symptom onset of infection, was also reported in Case 3. CSF analysis and PCR from Cases 2 and 3 were normal. Radiological evaluation of the chest was normal in Case 3 but revealed bilateral ground-glass opacities in Cases 1 and 2, which are consistently seen in COVID-19 pneumonia [3,37]. Brain CT or MRI was not done in Case 2, and both were normal in Case 1, but the brain MRI revealed enhancement of CN VII in Case 3 (Table 1).

**Table 1:** Diagnostic modalities utilized in the presented cases. RT-PCR: reverse transcriptase polymerase chain reaction; SARS-CoV-2: severe acute respiratory syndrome coronavirus 2; CXR: chest X-ray; CT: computed tomography; MRI: magnetic resonance imaging; N/A: not available; BGGO: bilateral ground glass opacities; CN VII: cranial nerve VII (facial nerve); HSV: herpes simplex virus; VZV: varicella zoster virus; CMV: cytomegalovirus; EBV: Epstein-Barr virus; CSF: cerebrospinal fluid; HIV: human immunodeficiency virus; EMG: electromyography; NVC: nerve conduction velocity; FNP: facial nerve palsy; OD: olfactory dysfunction; GD: gustatory dysfunction. Adapted from Refs. [31-33].

	Case 1	Case 2	Case 3
RT-PCR of SARS-CoV-2	Positive	Positive	Positive
Imaging results	CXR-N/A; Lung CT-BGGO; Brain CT-normal; Brain MRI-normal	CXR-normal; Lung CT-BGGO; Brain CT-N/A; Brain MRI-N/A	CXR-normal; Lung CT-N/A; Brain CT-N/A; Brain MRI-Enhancement of left CN VII
Serologies	Anti-SARS-CoV-2-Higher than upper normal limit; anti-HSV-Negative; anti- VZV-Negative	N/A	N/A
CSF analysis and other CSF tests	N/A	CSF pressure-20 cmH_2_O; Cell count-<1/μL; Total protein-17 mg/dL; Glucose-61 mg/dL; CSF RT-PCR of SARS-CoV-2-Negative	Normal CSF analysis; CSF PCR of HSV, VZV, CMV, EBV-Negative; CSF RT-PCR of SARS-CoV-2-Negative.
Other relevant tests	HIV antibody-negative; EMG and NCV-Abnormality of the left CN VII	Rapid flu-Negative; Rapid Strep-Negative	Kernig-Negative; Brudzinski-Negative
Neurological outcome at last follow-up	FNP partially improved but not resolved at one-month follow-up; OD and GD fully resolved. Patient then lost to follow-up.	FNP, anosmia, and dysgeusia completely resolved.	No significant change in FNP at one-week follow-up; headache and retroauricular pain improved.
Time to partial/complete recovery	OD/GD resolved by two weeks; FNP still incomplete at one month (no further data-lost to follow-up)	Symptoms gradually improved by day four; complete resolution by day 11	Not resolved as of one-week follow-up (no further follow-up reported)

Therapeutic approaches and the absence of guidelines

Therapeutically, Case 1 was prescribed dexamethasone and lopinavir/ritonavir (Kaletra), while Case 3 was prescribed prednisone, valacyclovir, and lopinavir/ritonavir (Kaletra) to inhibit SARS-CoV-2 replication. Although multiple reports have suggested that Kaletra is not an effective treatment for COVID-19 [38-40], one small, non-randomized cohort study suggested that it may reduce viral shedding duration relative to remdesivir, though this finding should be interpreted cautiously given the limited sample size and study design [40]. In Case 2, a Japanese Kampo medicine, Maoto, was used instead of steroids. No evidence-based treatment guideline currently exists for COVID-19-associated FNP; the heterogeneous regimens used across these three cases (corticosteroids, antivirals, and traditional Kampo medicine) reflect individualized clinical judgment rather than a validated protocol. 

Follow-up and Recovery

At the two-week follow-up in Case 1, complete resolution of anosmia and dysgeusia, as well as reduced facial weakness, was noted. At the one-month follow-up, the patient’s COVID-19 RT-PCR test was negative, and the FNP had further improved but was not yet completely resolved. Unfortunately, the patient was then lost to follow-up. In Case 2, the patient's FNP, anosmia, and dysgeusia gradually improved by day four, but their respiratory symptoms worsened concurrently. After additional treatment, her symptoms completely resolved by day 11. The patient was discharged on day 16 after two negative SARS-CoV-2 PCR tests. In Case 3, no notable changes were observed during the one-week follow-up; however, headache and retroauricular pain had improved.

The pooled risk ratio of FNP during the COVID-19 pandemic compared with the pre-pandemic period was reported to be 1.68 (95% CI: 1.16-2.43; p = 0.01) [41], and an estimated incidence of FNP during this time was ~82 per 100,000 patients [42]. The exact pathophysiology of OD, GD, and FNP in the context of COVID-19 infection remains elusive, and several potential pathophysiological explanations have been proposed based on multiple literature reviews.

Proposed pathophysiologic mechanisms

Several mechanisms have been proposed to explain the OD associated with COVID-19, including nasal obstruction, infection, or damage to olfactory receptor neurons; damage to olfactory centers in the brain; dysfunction of olfactory supporting cells; inflammation-related dysfunction of the olfactory epithelium [43,44]; and effects of zinc deficiency [45]. Among these potential mechanisms, anosmia associated with COVID-19 implicates damage to the olfactory epithelium, particularly sustentacular cells. This is supported by higher levels of ACE2 and transmembrane protease, serine 2 (TMPRSS-2) expression [46-49] than in other supporting cells, the corresponding regeneration time to the recovery of anosmia [43,44,50], and massive damage induced by SARS-CoV-2 in the olfactory epithelium by infecting sustentacular cells and not the olfactory sensory neurons [51].

The proposed mechanisms of GD associated with COVID-19 are collateral damage to receptors due to an overactive immune system [52], disruption of ACE2-associated taste perception, occupation of sialic acid binding sites of the salivary mucin, inability to perceive flavors due to concomitant presence of OD, zinc deficiency, and direct viral attack on several oral sites via ACE2 [43,53]. CN VII's special visceral afferent (SVA) component carries information about taste from the anterior two-thirds of the tongue [54]. Therefore, CN VII palsy can also lead to unilateral ageusia or dysgeusia.

The pathophysiology of FNP proposed in association with COVID-19 can be categorized into infectious, immune-mediated, or ischemic mechanisms. Infectious causes involve direct viral toxicity [55] and reactivation or co-infection with neurotropic viruses like HSV or VZV [56]. Evidence of neuronal damage without significant inflammation, as seen in SARS-CoV [57] and SARS-CoV-2 [58], and post-mortem findings of viral particles in the nerve axons [59] further support the former, whereas impaired immune response [56,60-62] and viral etiology of FNP [63] support the latter mechanism. Immune-mediated responses include autoimmune reactions like GBS or its variants [64,65], which are frequently associated with FNP [66,67]. Inflammation-induced demyelination is related to axonal diffusion, viral replication [68,69], and nerve damage due to an inflammatory feedback loop [52]. Ischemic causes include ischemia of the vasa nervorum [70] due to COVID-19-associated microthrombi [71] or edema, leading to compressive ischemia of the nerve [68]. The latter is especially true for CN VII due to its intratemporal course through a narrow and bony canal [63].

Across these three symptom domains, the strength of mechanistic evidence varies considerably. Direct evidence, such as post-mortem detection of viral particles within nerve axons [59] and demonstrated ACE2/TMPRSS-2 co-expression in olfactory sustentacular cells [46-49], supports a plausible route of epithelial or neural injury, particularly for OD. By contrast, several proposed mechanisms, including zinc deficiency, sialic acid receptor blockade, and an inflammatory feedback loop, remain highly inferential, based on biochemical possibility. The proposed mechanisms should not be mistaken for consensus on any single pathway. 

Association versus causation

Although an increased incidence of FNP has been observed during the COVID-19 pandemic, a definite causative relationship with SARS-CoV-2 remains unproven. The presented cases represent a small sample size, and the possibility that their FNP was idiopathic and unrelated to COVID-19 cannot be excluded. Prospective cohort studies or case-control designs directly comparing COVID-positive and COVID-negative patients presenting with FNP would help clarify whether this association reflects a true pathophysiological link or coincidental co-occurrence; a multinational case registry could also help address the persistent small-sample-size problem in this literature.

## Conclusions

The cases varied in clinical presentation, environmental risk factors, and diagnostic patterns, illustrating some of the diverse ways COVID-19 may affect the nervous system, though conclusions from three cases cannot be generalized to the full spectrum of presentations. Despite varied treatment modalities, partial to complete symptom remission was evident across all three cases, though the absence of a standardized protocol limits any inference about which treatment approach was responsible. The underlying mechanisms of cranial nerve involvement remain elusive, warranting further study to clarify potential viral, immune-mediated, and vascular contributions. More investigation is necessary to develop standardized diagnostic and treatment approaches to elucidate the connection between SARS-CoV-2 and cranial nerve damage.

## References

[1] (2025). WHO. COVID-19 cases, WHO COVID-19 dashboard. https://data.who.int/dashboards/covid19/cases.

[2] Asselah T, Durantel D, Pasmant E, Lau G, Schinazi RF (2021). COVID-19: discovery, diagnostics and drug development. J Hepatol.

[3] Baj J, Karakuła-Juchnowicz H, Teresiński G (2020). COVID-19: specific and non-specific clinical manifestations and symptoms: the current state of knowledge. J Clin Med.

[4] Vakili K, Fathi M, Hajiesmaeili M (2021). Neurological symptoms, comorbidities, and complications of COVID-19: a literature review and meta-analysis of observational studies. Eur Neurol.

[5] Liu J, Li Y, Liu Q (2021). SARS-CoV-2 cell tropism and multiorgan infection. Cell Discov.

[6] Shirbhate E, Pandey J, Patel VK (2021). Understanding the role of ACE-2 receptor in pathogenesis of COVID-19 disease: a potential approach for therapeutic intervention. Pharmacol Rep.

[7] Li MY, Li L, Zhang Y, Wang XS (2020). Expression of the SARS-CoV-2 cell receptor gene ACE2 in a wide variety of human tissues. Infect Dis Poverty.

[8] Zubair AS, McAlpine LS, Gardin T, Farhadian S, Kuruvilla DE, Spudich S (2020). Neuropathogenesis and neurologic manifestations of the coronaviruses in the age of coronavirus disease 2019: a review. JAMA Neurol.

[9] Doblan A, Kaplama ME, Ak S (2021). Cranial nerve involvement in COVID-19. Am J Otolaryngol.

[10] Ousseiran ZH, Fares Y, Chamoun WT (2023). Neurological manifestations of COVID-19: a systematic review and detailed comprehension. Int J Neurosci.

[11] Molaverdi G, Kamal Z, Safavi M (2023). Neurological complications after COVID-19: a narrative review. eNeurologicalSci.

[12] Long COVID (Post-COVID Conditions, PCC) > Fact Sheets > Yale Medicine (2025). Yale Medicine. Long COVID (post-COVID conditions, PCC), fact sheets. https://www.yalemedicine.org/conditions/long-covid-post-covid-conditions-pcc.

[13] Wesselingh R (2023). Prevalence, pathogenesis and spectrum of neurological symptoms in COVID-19 and post-COVID-19 syndrome: a narrative review. Med J Aust.

[14] Sharma A, Kumar A, Flora SJ (2021). Neurological manifestations in COVID-19 patients: a meta-analysis. ACS Chem Neurosci.

[15] Jagst M, Pottkämper L, Gömer A, Pitarokoili K, Steinmann E (2024). Neuroinvasion and neurotropism of severe acute respiratory syndrome coronavirus 2 infection. Curr Opin Microbiol.

[16] Bauer L, Laksono BM, de Vrij FM, Kushner SA, Harschnitz O, van Riel D (2022). The neuroinvasiveness, neurotropism, and neurovirulence of SARS-CoV-2. Trends Neurosci.

[17] Hernández-Parra H, Reyes-Hernández OD, Figueroa-González G (2023). Alteration of the blood-brain barrier by COVID-19 and its implication in the permeation of drugs into the brain. Front Cell Neurosci.

[18] Pontelli MC, Castro ÍA, Martins RB (2022). SARS-CoV-2 productively infects primary human immune system cells in vitro and in COVID-19 patients. J Mol Cell Biol.

[19] Pellegrini L, Bonfio C, Chadwick J, Begum F, Skehel M, Lancaster MA (2020). Human CNS barrier-forming organoids with cerebrospinal fluid production. Science.

[20] Pacheco-Herrero M, Soto-Rojas LO, Harrington CR (2021). Elucidating the neuropathologic mechanisms of SARS-CoV-2 infection. Front Neurol.

[21] Ong WY, Satish RL, Herr DR (2022). ACE2, circumventricular organs and the hypothalamus, and COVID-19. Neuromolecular Med.

[22] Generoso JS, Barichello de Quevedo JL, Cattani M (2021). Neurobiology of COVID-19: how can the virus affect the brain?. Braz J Psychiatry.

[23] Fenrich M, Mrdenovic S, Balog M (2020). SARS-CoV-2 dissemination through peripheral nerves explains multiple organ injury. Front Cell Neurosci.

[24] Zhou Z, Kang H, Li S, Zhao X (2020). Understanding the neurotropic characteristics of SARS-CoV-2: from neurological manifestations of COVID-19 to potential neurotropic mechanisms. J Neurol.

[25] Sleigh JN (2020). Axonal transport: the delivery system keeping nerve cells alive. Front Young Minds.

[26] Jha NK, Ojha S, Jha SK (2021). Evidence of coronavirus (CoV) pathogenesis and emerging pathogen SARS-CoV-2 in the nervous system: a review on neurological impairments and manifestations. J Mol Neurosci.

[27] Tonkal A, Alamri AA, AlMaghrabi SJ (2022). Cranial nerve impairment associated with COVID-19 infections: a systematic review. Cureus.

[28] Finsterer J, Scorza FA, Scorza C, Fiorini A (2022). COVID-19 associated cranial nerve neuropathy: a systematic review. Bosn J Basic Med Sci.

[29] Hanganu AR, Constantin A, Moise ES (2023). Peripheral nervous system involvement associated with COVID-19. A systematic review of literature. PLoS One.

[30] Heidari ME, Nazemi P, Feizabad E, Beiranvand F, Afzali M (2023). Cranial nerve involvement among COVID-19 survivors. Front Neurol.

[31] Afshar ZM, Babazadeh A, Afsharian M, Vaziri S, Ebrahimpour S (2021). Bell’s palsy associated with COVID-19 infection: a case report. Oman Med J.

[32] Homma Y, Watanabe M, Inoue K, Moritaka T (2020). Coronavirus disease-19 pneumonia with facial nerve palsy and olfactory disturbance. Intern Med.

[33] Goh Y, Beh DL, Makmur A, Somani J, Chan AC (2020). Pearls & Oy-sters: facial nerve palsy in COVID-19 infection. Neurology.

[34] Sheikh Z., Silverstone L., & Sharma R. (2015 (2025). Radiopaedia.Org. Facial palsy. https://radiopaedia.org/articles/facial-palsy?lang=gb.

[35] Rubin M. (2023, November 2 (2025). MSD Manual Professional Edition. Facial nerve palsy. https://www.msdmanuals.com/professional/neurologic-disorders/neuro-ophthalmologic-and-cranial-nerve-disorders/facial-nerve-palsy.

[36] Holland NJ, Weiner GM (2004). Recent developments in Bell's palsy. BMJ.

[37] Churruca M, Martínez-Besteiro E, Couñago F, Landete P (2021). COVID-19 pneumonia: a review of typical radiological characteristics. World J Radiol.

[38] Horby PW, Mafham M, Bell JL (2020). Lopinavir-ritonavir in patients admitted to hospital with COVID-19 (RECOVERY): a randomised, controlled, open-label, platform trial. Lancet.

[39] Cao B, Wang Y, Wen D (2020). A trial of lopinavir-ritonavir in adults hospitalized with severe COVID-19. N Engl J Med.

[40] Kim CM, Chung JK, Tamanna S (2024). Comparable efficacy of lopinavir/ritonavir and remdesivir in reducing viral load and shedding duration in patients with COVID-19. Microorganisms.

[41] Mirza AA, Almalki AH, Noori FA, Neazy SA, Dahm V, Bajin MD, Lin VY (2025). Facial nerve palsy amid the SARS-CoV-2 pandemic: a pooled analysis. J Otolaryngol Head Neck Surg.

[42] Tamaki A, Cabrera CI, Li S, Rabbani C, Thuener JE, Rezaee RP, Fowler N (2021). Incidence of Bell palsy in patients with COVID-19. JAMA Otolaryngol Head Neck Surg.

[43] Mutiawati E, Fahriani M, Mamada SS (2021). Anosmia and dysgeusia in SARS-CoV-2 infection: incidence and effects on COVID-19 severity and mortality, and the possible pathobiology mechanisms-a systematic review and meta-analysis. F1000Research.

[44] Butowt R, von Bartheld CS (2021). Anosmia in COVID-19: underlying mechanisms and assessment of an olfactory route to brain infection. Neuroscientist.

[45] Ahmed AK, Sayad R, Mahmoud IA (2022). "Anosmia" the mysterious collateral damage of COVID-19. J Neurovirol.

[46] Klingenstein M, Klingenstein S, Neckel PH (2020). Evidence of SARS-CoV2 entry protein ACE2 in the human nose and olfactory bulb. Cells Tissues Organs.

[47] Bilinska K, Jakubowska P, Von Bartheld CS, Butowt R (2020). Expression of the SARS-CoV-2 entry proteins, ACE2 and TMPRSS2, in cells of the olfactory epithelium: identification of cell types and trends with age. ACS Chem Neurosci.

[48] Brann DH, Tsukahara T, Weinreb C (2020). Non-neuronal expression of SARS-CoV-2 entry genes in the olfactory system suggests mechanisms underlying COVID-19-associated anosmia. Sci Adv.

[49] Fodoulian L, Tuberosa J, Rossier D (2020). SARS-CoV-2 receptors and entry genes are expressed in the human olfactory neuroepithelium and brain. iScience.

[50] Schwob JE (2002). Neural regeneration and the peripheral olfactory system. Anat Rec.

[51] Bryche B, St Albin A, Murri S (2020). Massive transient damage of the olfactory epithelium associated with infection of sustentacular cells by SARS-CoV-2 in golden Syrian hamsters. Brain Behav Immun.

[52] Carvajal JJ, García-Castillo V, Cuellar SV (2024). New insights into the pathogenesis of SARS-CoV-2 during and after the COVID-19 pandemic. Front Immunol.

[53] Vaira LA, Salzano G, Fois AG, Piombino P, De Riu G (2020). Potential pathogenesis of ageusia and anosmia in COVID-19 patients. Int Forum Allergy Rhinol.

[54] Dulak D, Naqvi IA (2025). Neuroanatomy, cranial nerve 7 (facial). StatPearls (Internet).

[55] Martins AS, Losa FJ, Rueda HH, García-Gasalla M (2021). Facial nerve palsy as a neurological manifestation of COVID-19. J Glob Infect Dis.

[56] Kim JY, Ragusa M, Tortosa F (2023). Viral reactivations and co-infections in COVID-19 patients: a systematic review. BMC Infect Dis.

[57] Baig AM, Khaleeq A, Ali U, Syeda H (2020). Evidence of the COVID-19 virus targeting the CNS: tissue distribution, host-virus interaction, and proposed neurotropic mechanisms. ACS Chem Neurosci.

[58] Hsu JC, Saenkham-Huntsinger P, Huang P (2024). Characterizing neuroinvasion and neuropathology of SARS-CoV-2 by using AC70 human ACE2 transgenic mice. Front Microbiol.

[59] Matschke J, Lütgehetmann M, Hagel C (2020). Neuropathology of patients with COVID-19 in Germany: a post-mortem case series. Lancet Neurol.

[60] Yang M, Lin C, Wang Y (2022). Cytokine storm promoting T cell exhaustion in severe COVID-19 revealed by single cell sequencing data analysis. Precis Clin Med.

[61] Zheng M, Gao Y, Wang G (2020). Functional exhaustion of antiviral lymphocytes in COVID-19 patients. Cell Mol Immunol.

[62] Diao B, Wang C, Tan Y (2020). Reduction and functional exhaustion of T cells in patients with coronavirus disease 2019 (COVID-19). Front Immunol.

[63] Walker NR, Mistry RK, Mazzoni T (2025). Facial nerve palsy. StatPearls (Internet).

[64] Valaparla VL, Rane SP, Patel C, Li X (2024). Guillain-Barre syndrome and link with COVID-19 infection and vaccination: a review of literature. Front Neurol.

[65] Pimentel V, Luchsinger VW, Carvalho GL (2023). Guillain-Barré syndrome associated with COVID-19: a systematic review. Brain Behav Immun Health.

[66] Bhargava A, Banakar BF, Pujar GS, Khichar S (2014). A study of Guillain-Barré syndrome with reference to cranial neuropathy and its prognostic implication. J Neurosci Rural Pract.

[67] Andary M. T., Oleszek J. L., Maurelus K., & White-McCrimmon R. Y. (2024, October 29 (2025). Medscape. Guillain-Barre syndrome clinical presentation. https://emedicine.medscape.com/article/315632-clinical#b3.

[68] Zhang W, Xu L, Luo T (2020). The etiology of Bell's palsy: a review. J Neurol.

[69] Perilli L, Fetta M, Capponi M (2023). Peripheral nervous system involvement in SARS-CoV-2 infection: a review of the current pediatric literature. Front Neurol.

[70] Lima MA, Silva MT, Soares CN (2020). Peripheral facial nerve palsy associated with COVID-19. J Neurovirol.

[71] McFadyen JD, Stevens H, Peter K (2020). The emerging threat of (micro) thrombosis in COVID-19 and its therapeutic implications. Circ Res.

